# Generalizable Layered Blockchain Architecture for Health Care Applications: Development, Case Studies, and Evaluation

**DOI:** 10.2196/19029

**Published:** 2020-07-27

**Authors:** Yan Zhuang, Yin-Wu Chen, Zon-Yin Shae, Chi-Ren Shyu

**Affiliations:** 1 Institute for Data Science and Informatics University of Missouri - Columbia Columbia, MO United States; 2 Artifical Intelligence Research Lab Asia University Taichung Taiwan

**Keywords:** blockchain, smart contract, health information exchange, electronic health records, health care application

## Abstract

**Background:**

Data coordination across multiple health care facilities has become increasingly important for many emerging health care applications. Distrust has been recognized as a key barrier to the success of such applications. Leveraging blockchain technology could provide potential solutions tobuild trust between data providers and receivers by taking advantage of blockchain properties such as security, immutability, anonymity, decentralization, and smart contracts. Many health technologies have empirically proven that blockchain designs fit well with the needs of health care applications with certain degrees of success. However, there is a lack of robust architecture to provide a practical framework for developers to implement applications and test the performance of stability, efficiency, and scalability using standard blockchain designs. A generalized blockchain model is needed for the health care community to adopt blockchain technology and develop applications in a timely fashion.

**Objective:**

This study aimed at building a generalized blockchain architecture that provides data coordination functions, including data requests, permission granting, data exchange, and usage tracking, for a wide spectrum of health care application developments.

**Methods:**

An augmented, 3-layered blockchain architecture was built on a private blockchain network. The 3 layers, from bottom to top, are as follows: (1) incorporation of fundamental blockchain settings and smart contract design for data collection; (2) interactions between the blockchain and health care application development environment using Node.js and web3.js; and (3) a flexible development platform that supports web technologies such as HTML, https, and various programing languages. Two example applications, health information exchange (HIE) and clinical trial recruitment, were developed in our design to demonstrate the feasibility of the layered architecture. Case studies were conducted to test the performance in terms of stability, efficiency, and scalability of the blockchain system.

**Results:**

A total of 331,142 simulated HIE requests from accounts of 40,000 patients were successfully validated through this layered blockchain architecture with an average exchange time of 11.271 (SD 2.208) seconds. We also simulated a clinical trial recruitment scenario with the same set of patients and various recruitment criteria to match potential subjects using the same architecture. Potential subjects successfully received the clinical trial recruitment information and granted permission to the trial sponsors to access their health records with an average time of 3.07 seconds.

**Conclusions:**

This study proposes a generalized layered blockchain architecture that offers health technology community blockchain features for application development without requiring developers to have extensive experience with blockchain technology. The case studies tested the performance of our design and empirically proved the feasibility of the architecture in 2 relevant health application domains.

## Introduction

### Background

The health care industry generates abundant health data from various sources [1]. The meaningful use of health data can improve the decisions of health care providers and patient outcomes [2]. The increasing adoption of digitalized health care records such as electronic health records (EHRs) provides the opportunity for health care data analytics and the coordination of results with care of patients [3,4]. Many health care applications are designed to maximize the potential benefits of EHR usage, such as analyzing epidemiological disease patterns to improve public health across the nation or timely health information exchange (HIE) to provide patients with coordinated and efficient care across health care facilities. Data sharing is needed when required data are distributed and stored in different sources [5,6]. However, multiple barriers to data coordination exist: (1) data privacy and security concerns during HIE [7,8], (2) the limitations of institutional privacy rules [3,9], and (3) the time-consuming process of generating agreements on data exchange between institutions [10,11]. There are security and privacy concerns about the exchange of sensitive health data [8]. Due to the Health Insurance Portability and Accountability Act, legislation limits EHR access without patient authorization [3,12]. Therefore, there needs to be a sustainable and secure data collection mechanism by which each data owner can maintain control of their data and only if the owner of the data allows it to occur [13].

### Blockchain Approaches

Facing the challenges of data coordination, blockchain is considered to be a disruptive technology that fits the needs of many health care applications [14,15]. Blockchain is a distributed ledger technology that was first applied in the financial sector [16]. Bitcoin is one of the most popular applications of blockchain that shows its security, durability, and robustness. All the users in the blockchain are anonymous and represented by unique pairs of randomly generated 256-bit public and private keys [16]. This feature of protecting user privacy is one of the reasons for the success of Bitcoin and is also why blockchain is considered for potential health care applications without concerns for patient privacy issues [17]. Similar to the public blockchain used for Bitcoin, blockchain can be implemented privately, also known as a *permissioned chain* for different applications [18]. Users need to gain permission to join the private chain, which limits the data access of the blockchain to only authorized users. Blockchain is a chain of blocks that contain current and former block numbers and validated transactions occurring in a short period [19]. Generation of blocks follows a certain consensus protocol, such as Proof of Work (PoW), which requires miners to provide computing power to validate the legitimacy of the transactions and generate new blocks. Data owners can use blocks to track the timestamps and requesters of their data. All transactions are publicly auditable. Any malicious transaction is expected to be detected by the users in the blockchain and will be discarded thereafter [20]. Once a transaction is made, all the users will validate the identity of the sender and the legitimacy of the transaction. There is no trusted third party to perform the validation process. Rather, the legitimacy of the transaction data accuracy and data provenance is ensured by all the users in a transparent manner [21]. Verified transactions will be written into the subsequent block, and the contents of the transaction can never be erased or changed [16]. The Ethereum blockchain keeps all the original blockchain features and adds a function of programmable self-executing computer protocols to the blockchain system with agreements between the requester and receiver called *smart contracts* [19]. Smart contracts are coded in Turing-complete languages such as Solidity in the Ethereum blockchain that can solve any computational problems [22]. For example, using smart contracts can regulate transactions such as enforcing the interoperability standard of exchanged data [23].

As the features of decentralized transaction validation—ensured data provenance, data sharing, data integration, and flexibility of smart contracts—fit well with the needs of many health care applications, there have been many efforts to apply blockchain to areas of health care, such as HIE, pharmaceutical supply chains, and clinical trial management [24,25]. However, most blockchain applications in the health care area are still in the early stages of implementation [15]. 

### Objectives

**Figure 1:**
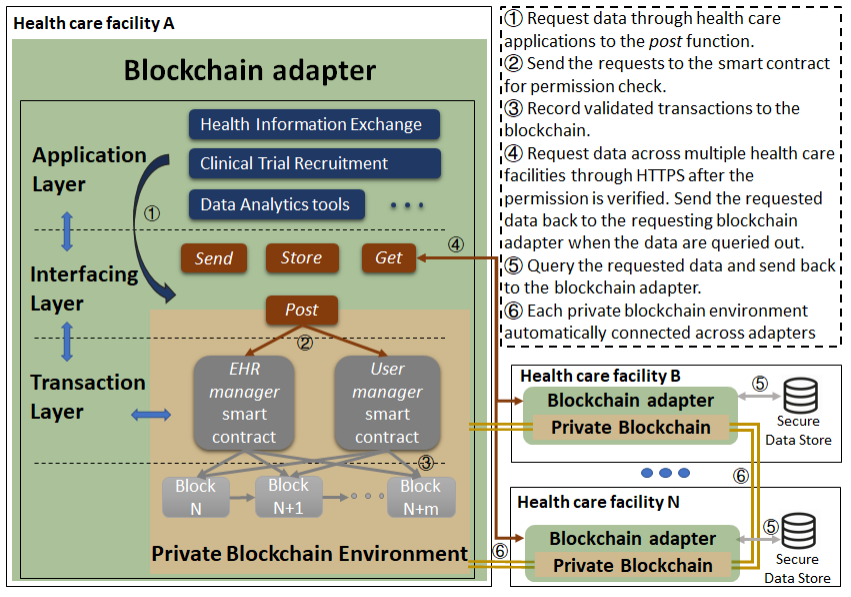
Overall layered blockchain architecture. The transaction layer consists of 2 smart contracts to manage data access tasks. The interfacing layer interacts with the blockchain environment, graphical user interfaces, and other blockchain adapters. The application layer provides a flexible platform for health care application development. The figure shows the general process of data requests using the architecture. EHR: electronic health record.

This was an extended study of previous blockchain application designs and an exploration of a new generalized layered architecture, as shown in Figure 1. We built a private blockchain environment, implemented the layered architecture, and built 2 prototype applications based on this architecture: HIE and subject recruitment for clinical trials.

Compared with traditional blockchain architecture, this study proposed a generalized blockchain system that fits a wide spectrum of health care applications for cross-site data coordination. The layered architecture provides a blockchain platform with predefined functions for data collection for developers to implement health care applications without an extensive experience of blockchain. We explained the blockchain environment setup and the new layered architecture in the Methods section, followed by case studies and simulation results in the Results section to demonstrate the feasibility, scalability, and compatibility of the architecture for health care application development.

## Methods

### Augmented Three-Layered Blockchain Architecture

The blockchain network offers advantages in managing digital assets [26,27]. A well-known digital asset managed by blockchain is Bitcoin or, in general, cryptocurrency [25]. In health care applications, EHR access is a digital asset in management. The health data for patients who opt to participate in the blockchain are encrypted and stored in secured off-blockchain databases located in health care facilities protected by their own firewalls. The foundation private blockchain is used to store all transactions referring to EHR requests and exchanges and the metadata that contain pertinent health care data with the identifications of patients and health care facilities. In addition, the following components are captured in the metadata: time of creation, data set location, access permission and control, data decryption, and data authenticity.

As blockchain is a fully distributed system, we have built 3 layers on the top of the private blockchain network: *transaction layer*, *interfacing layer*, and *application layer.* As shown in Figure 1, the *transaction layer* consists of 2 types of smart contracts coded in Solidity, namely, *EHR manager* smart contracts and *user manager* smart contracts, to manage the storage of and access to metadata that are encrypted with decentralized validity and authenticity checks using blockchain security [28]. These 2 smart contracts are fixed in the system and are not permitted to change.

Data requests from one health facility to another trigger certain functions of smart contracts through the *interfacing layer*. Only through the information in the trustable metadata can original data be retrieved and verified for authenticity by the *interfacing layer*. Applications such as subject recruitment for clinical trials, EHR management, and artificial intelligence (AI)–based data analytics tools can be built on the *application layer*. This layered architecture has the following benefits compared with the previous blockchain systems used for health care applications: (1) compatibility of most health care applications that require data exchange, (2) semipublicity to fix the blockchain settings and smart contract functions but retain most blockchain features, (3) security settings of each layer to protect the identities and data of patients during an exchange, and (4) traceability of who have accessed the data and how they used the data.

### Environment Setup

To build the blockchain system, each health care facility is required to provide at least one blockchain node, which is an electronic device that can install the blockchain system. In our setting, each node runs the official Go implementation of the Ethereum protocol on an Ubuntu Linux server. To connect the blockchain with EHR systems, we have developed a blockchain adapter, which is a blockchain node designed to abide by the local data access policies set by individual health care facilities [29].

To ensure the security of health care data and meet the needs of current EHR operations, our blockchain system does not store patient data. There are two main reasons for this. First, it is not practical to store a large chunk of health care data in a blockchain because of the health care facilities’ policies of sharing health information and blockchain storage constraints [25]. Second, the health care industry is still unreceptive to allowing patient data to move across the blockchain network [12]. A metadata set containing pertinent information of the original EHR data is created and submitted to the blockchain platform. The creation and updating of metadata are recorded into a chain of data blocks in the blockchain. These transactions executed via smart contracts are immutable and traceable, thus creating a trustable metadata transaction. As there are different interoperability standards, such as Fast Healthcare Interoperability Resources and Health Level 7 version 3, there will be different metadata points of different data stores for the receiver to choose from. The receiver can choose the compatible interoperability standard of their home department’s standard during the HIE process [30,31]. The metadata owner, who is the same as the data set owner, can grant, reject, or revoke access permission automatically via smart contract or interactively by means of electronic notification and confirmation. For example, smart contracts can be programmed to grant or reject access permission based on time or data type or to delegate access permission to a specific user. In all cases, because a third-party intervention is not necessary for granting or rejecting permissions, the time efficiency of data sharing can be greatly improved. This is the decentralized feature of the blockchain network that enables peer-to-peer HIE.

Each blockchain user, including the blockchain adapter, is represented by a hash value (account address) derived from the public key generated by the blockchain [32]. The private key is kept private by the user, and the public key is used for internal and external transactions and communications. Any transaction related to the account address must be signed by the signature, which is the private key. All transactions need to ensure the public key and the private key matched before transactions are recorded in the blockchain. The patients need to go to the health care facilities to opt into the system so that they can claim ownership of their data. A user can create a username and password or use biometric information that is mapped to the public and private key instead of memorizing the real key’s value. The metadata permission control carried out by smart contract is anonymous, which ensures privacy. The metadata used for locating encrypted data are communicated with the secured data stores via the https protocol, and the result is communicated back to the user via the same protocol; thus, it is considered to be a secure data transfer.

### Foundation Private Blockchain Network

The foundation of our layered architecture is a private Ethereum blockchain, which involves an immutable chain of data blocks consisting of committed ledgers and multiple blockchain nodes synchronously maintaining the same chain of data blocks. In the overall architecture, this layer ensures data immutability, decentralized consensus, data transparency, and traceability. The private blockchain is initiated from a starting node with special settings to make the blockchain unique. The smart contracts are deployed through the starting node when the private blockchain is built. All the participating nodes from health care facilities must obtain permission from the starting node to join the system. This procedure will disallow unauthorized parties from joining the system. As the participating nodes joined into the system, the blockchain will automatically generate accounts for their blockchain adapters. All other users, such as patients and health care providers, need to opt into the system through health care facilities. The blockchain accounts will be generated for the users from each health care facility as soon as the applicants’ identities are proven.

The private blockchain stores all the transactions for (1) patients and health care facilities granting, revoking, and denying access to their EHR data; (2) authentication of patients and health care providers to retrieve the EHR data; and (3) health care facilities to store metadata for patient visits. The transactions will record the receiver, sender, contained data, and the timestamp into the blocks through blockchain adapters. Users can also make transactions in the backend blockchain console through the blockchain node. These transactions still need to pass smart contract regulations to become effective. Most users will interact with the graphical user interfaces (GUIs) built on the *application layer* to execute functions in the blockchain system.

### Transaction Layer

The *transaction layer* consists of 2 smart contracts that specify a metadata model for health care records and methods that regulate data access rights, permission policies, and data encryption. Two smart contracts, the *EHR manager* smart contract and the *user manager* smart contract, are deployed to the blockchain to securely accomplish the basic EHR management tasks. The *EHR manager* smart contract can only be used by health care facilities to submit the EHR metadata to the blockchain. The *user manager* smart contract is used by patients or facilities to manage access to their data. Once a patient has opted into the system from a health care facility, the health care facility’s blockchain adapter will automatically encrypt his or her patient ID and public key using the patient’s private key and input to the *user manager* smart contract. Health care facilities will have adapter IDs stored in the *user manager* smart contract. The following scenarios demonstrate the use of smart contracts for the EHR metadata input and HIE.

#### Electronic Health Record Manager Smart Contract for Submitting Metadata to the Blockchain

**Figure 2:**
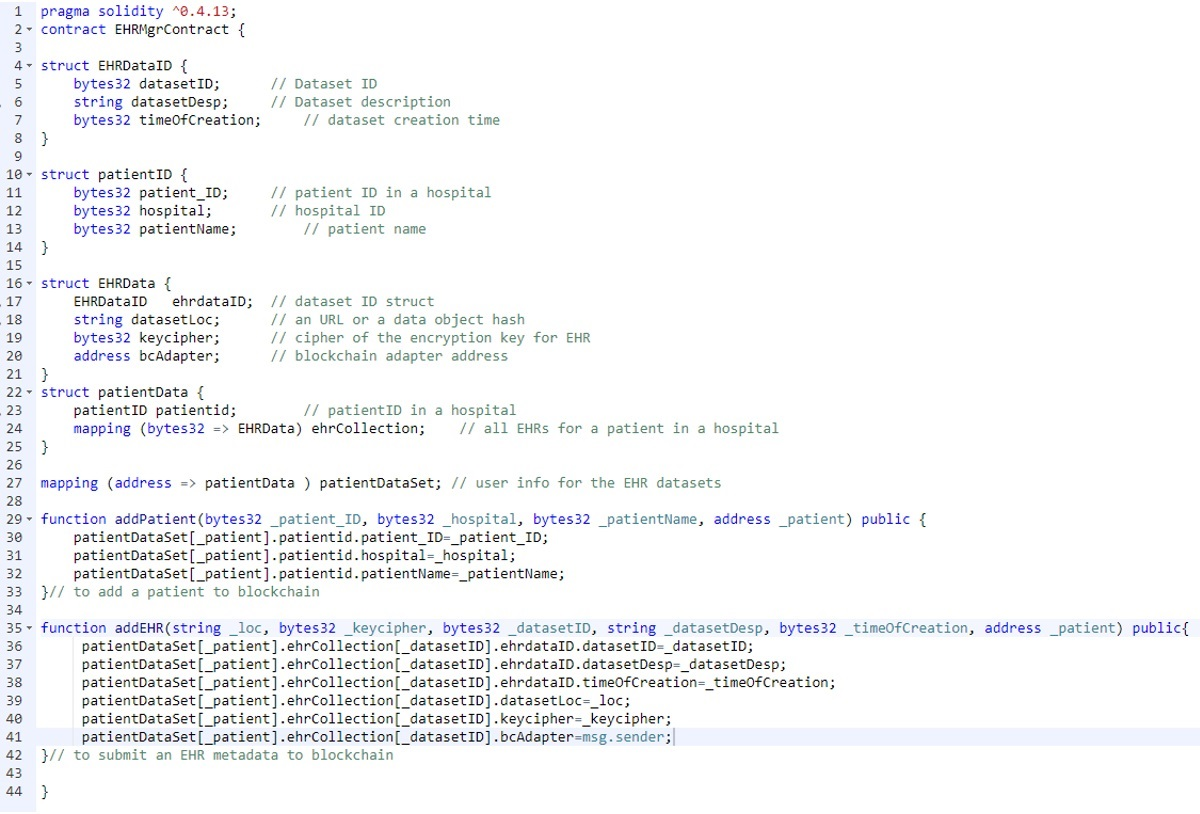
Main part of the electronic health record manager smart contract code that defines the metadata structure. Blockchain adapters must extract the information and calculate the encrypt keys and then store them into the smart contract. The record will automatically associate with the adapter’s blockchain ID.

The *EHR manager* smart contract (as shown in Figure 2) defines several structures to record patient information: EHRDataID and EHRdata define the metadata components, PatientID stores the patient ID and health care facility ID for the registered patients, and patientData maps the different health care facility visit records of patients with the patients’ IDs. Once the blockchain adapter receives a record from the EHR system, the blockchain adapter automatically performs the following steps to submit the metadata to the blockchain through an addEHR function:

1. Extract the patient ID from the EHR data set.
2. Find a public key associated with the patient from the *user manager* smart contract.
3. Generate a random *data key* for encrypting the EHR data set.
4. Encrypt the EHR data set using data key and store the encrypted data to an *off-chain* secured data store.
5. Use the patient’s public key to encrypt the data key. Call the encrypted data key “key cipher.”
6. Submit the following metadata to the blockchain:
  - Patient ID.
  - Encrypted data set location as a URL.
  - Key cipher.
  - Associated blockchain adapter ID.

**Figure 3:**
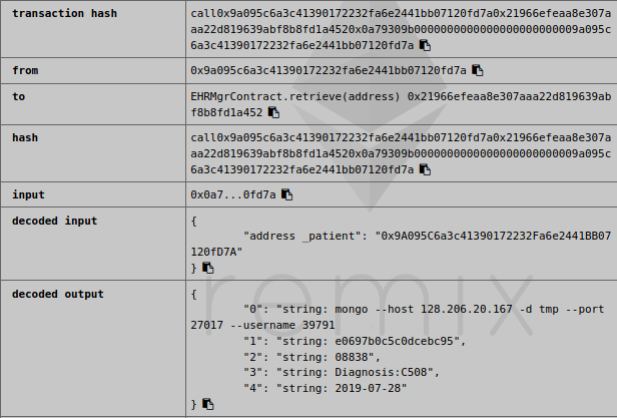
Example of a patient’s metadata retrieved through blockchain. The information is decoded by the Remix web-based integrated development environment, which is connected to the retrieving blockchain node. The patient’s metadata contain data location, key cipher, data set ID, data set description, and creation time.

The blockchain adapters will generate public and private key pairs following the Diffie-Hellman protocol using Node.js. The data encryption and decryption settings ensure that the data belonging to the patient is only decrypted by the health care facility that produced the data. The key cipher makes the data key more secure and can only be computed when the data owner is known. Figure 3 shows one patient’s decoded metadata retrieved through blockchain using the Remix integrated development environment, which is an open-source visualization tool used for interacting with blockchain nodes and smart contract development and deployment.

#### User Manager Smart Contract for the Health Information Exchange Process

A scenario is described in this section to show the process of health care provider X retrieving patient A’s EHR. Through a mobile application or web browser with biometric authentication, Patient A can grant access privileges to health care provider X using the application program interface (API) to set data permission on the *interface layer*. In some cases, more than one clinician is involved in the patient’s care. The health care facility needs to create a shared blockchain account for the provider’s department so that all involved clinicians can access the patient’s data with one-time authentication [33]. Blockchain adapters will record who has accessed the data and submit it to the blockchain. The blockchain adapters from the receiver’s home health care facility will perform the following steps for the data retrieval process:

1. Verify health care provider X’s permission to access patient A’s records through the *EHR manager* smart contracts.
2. Retrieve patient A’s metadata from the *EHR manager* smart contract.
3. Request the encrypted EHR data set from the remote health care facility via an https service provided by blockchain adapter.
4. Retrieve the encrypted data using encrypted data set location information in EHR metadata.
5. Decrypt the EHR data set. This step involves decrypting the key cipher using the patient’s private key to obtain the data key that decrypts the EHR data set.

Similar processes will be used for sharing data between health care facilities and for patients retrieving their own EHR records. The entire process will be performed automatically through the blockchain adapter.

### Interfacing Layer

The *interfacing layer* provides 4 high-level methods: *get* the health care data from different facilities, *store* the encrypted data securely, *post* metadata or data request to the blockchain via smart contracts in the *transaction layer*, and *send* the encrypted data to the receiver who has gained permission from the data owner. Using the functions in this layer, application developers can implement distributed data applications (DApps) without the knowledge of smart contracts and the underlying blockchain network. This layer consists of APIs and https web services to define a set of primitive coordinate functions: (1) submit data, (2) set data permission, and (3) retrieve data. The data submission API will extract metadata from the original data and call the *transaction layer*’s smart contracts to record it to the blockchain. It will also encrypt the original data and store the encrypted version to a secure off-blockchain data store. The data retrieval API will call smart contracts to retrieve metadata from blockchain, verify encrypted data authenticity with metadata, and decrypt the encrypted data in the off-blockchain data store to obtain the original data. The data permission setting API will call the *transaction layer*’s smart contracts to set access policies and methods for a piece of metadata. Information contained in metadata is used to retrieve and decrypt data. The https web services provide secure data transport when data are to be transported through a channel and can potentially be eavesdropped. Using the blockchain adapter to serve as a gateway to the EHR system minimizes the concerns of data exchange security.

**Figure 4:**
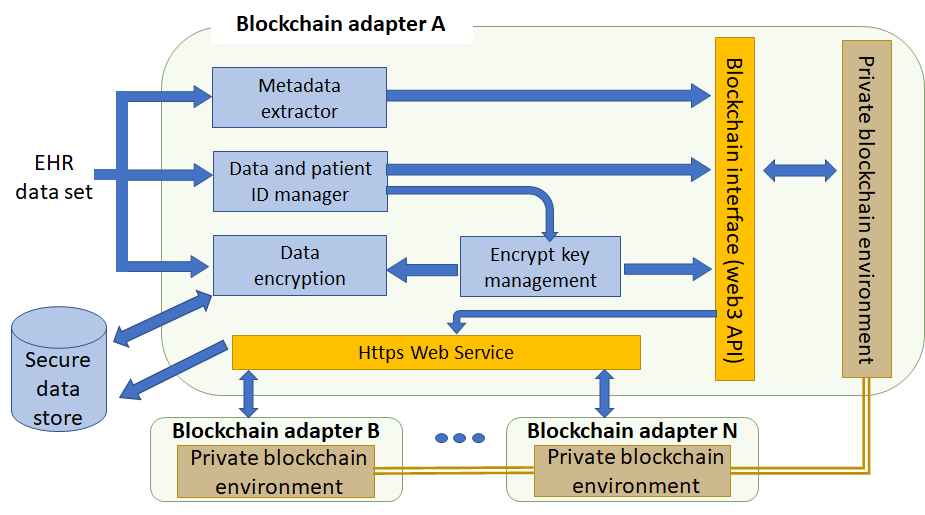
Blockchain adapter components and functions. Blockchain adapter extracts the metadata from the electronic health record, encrypts the electronic health record, stores the encrypted electronic health record into the secured data store, and maps the patient ID and data ID to the blockchain account. Blockchain adapters use https to interact with other adapters and communicate with the foundation private blockchain network through a blockchain interface. API: application program interface; EHR: electronic health record.

We implemented blockchain adapters as a Node.js application and used the web3.js package for interfacing with a blockchain node and https.js package for https secure web services. web3.js is also available in the Python library as web3.py. The https-based web services are mainly used for communication among blockchain adapters. The blockchain adapter is embedded as software that will install the missing component automatically, such as node.js and web3.js. Figure 4 shows a high-level block diagram of a blockchain adapter. The metadata extractor extracts metadata such as patient ID and data set ID from the EHR data set for data identification purposes in the blockchain. The data and patient ID manager maps the patient ID to the data set ID and records the information in both the *user manager* smart contract as well as the *EHR manager* smart contract. The data set encryption block in Figure 4 encrypts the EHR data set and stores away in the secure data store with a URL or a data object hash for future access.

From the security and reliability point of view, the following design guidelines are strictly followed for the blockchain adapter:

1. Blockchain adapter is modeled as a nonhuman blockchain user and has its own private and public key pair when the blockchain account or address is established. The public key is made public via the *user manager* smart contract. The private key is kept in a blockchain adapter.
2. The https service uses a separate key and certificate file.
3. The Diffie-Hellman key agreement protocol is used for data set encryption.
4. When one blockchain adapter fails, transactions (data set exchange) with the associated organization will be interrupted, but transactions among all other organizations will not be affected.

### Application Layer

With the above *interfacing layer* architecture and smart contract setup, many health care applications involving data exchange can be developed in the *application layer*. This layer will rely on the *interfacing layer* to securely collect the data and then perform data analytics. Applications will not change the existing blockchain settings. These applications can allow researchers or data owners to have better use of the EHR data. For example, personal health records management can be developed on the *application layer*. A patient’s identity will be verified through smart contracts in the blockchain. All patient records can be retrieved through the *interfacing layer*. In addition to the HIE application, subject recruitment for clinical trials could also be developed in this layer. Clinical trial sponsors need to obtain permission from the patients through blockchain adapters from clinical trial sites before the matching process [34]. After the patients grant the sponsors permission to gather their data, clinical trial sponsors can use the data analytics tools developed in the *application layer* to match the patients with their recruitment criteria automatically. We implemented these 2 sample applications on our private blockchain system. The Results section shows the interactions of the *application layer* and the blockchain system and the simulation results.

## Results

### Case Studies

To test the feasibility of our layered architecture, we built a blockchain environment that contains 1 starting node and 4 health care facility nodes. All the nodes have installed an Ubuntu 18.04.2 operating system and Go Ethereum with the default PoW setting. A blockchain adapter has been installed to each node to communicate with the blockchain and its own secured data store, which was implemented using MongoDB. We created 100 accounts for health care providers and 10,000 patient accounts on each health care facility node of patients’ records from the Surveillance, Epidemiology, and End Results database [35]. A total of 2431, 2587, and 2505 patients have multiple records distributivity stored in 2, 3, and 4 health care facility nodes, respectively. The remaining patients’ records were stored in a single facility node. Selected records were stored in each secured data store using an automated script following the same procedure described in the *EHR manager* smart contract. The ownerships have been claimed when the metadata were pushed into the blockchain using the smart contract in the *transaction layer*.

After setting up the environment, we implemented 2 applications to interact with our blockchain system. The applications are built off-chain but can communicate with the blockchain system using node.js. These 2 applications are examples of the potential use of the layered blockchain architecture. We tested the accuracy, scalability, and speed of our system. We made the following assumptions to simulate the 2 processes: (1) each clinical site has provided at least one node to the system, (2) each clinical site agrees to connect the blockchain adapter to the secured data store, and (3) patients have authorized the blockchain system as well as the 2 applications to access their health records.

### Health Information Exchange

This application provides an interface for users to manage access to personal health care records through the *transaction layer*. Patients can use this application to grant and revoke access to their records. Patients can also track how many times their records have been accessed through this aplication. To test the accuracy, speed, and scalability of our system, we simulated the process of patients granting permission to health care providers of their EHR. We developed 5 scripts to automate the simulation process by (1) randomly selecting 1 patient to grant 1 health care provider permission to their EHR per second for an hour and recording the timestamp, (2) recording the timestamp when the health care providers received the permission, (3) recording the timestamp when the health care providers received the data, and (5) adding 1 patient per second to script 1 then repeating scripts 1 to 4 until reaching the system limitation because of the known scalability constraints of Ethereum [36].

The simulation only contains the period of interactions with the blockchain. Retrieving data is an off-chain process through the https portal and varies with different health care facilities. From our simulation results, the system breaks at a certain point when the scale reaches 14 transactions per second (TPS). We simulated 331,142 access-granting transactions. All the transactions have successfully retrieved the records except the last second’s 14 transactions due to reaching the Ethereum scalability limitation. The average time of writing a transaction to a block is 11.271 (SD 2.208) seconds. We did not find a correlation between TPS and validation time. All health care providers received the metadata in an average of 1.73 seconds.

**Figure 5:**
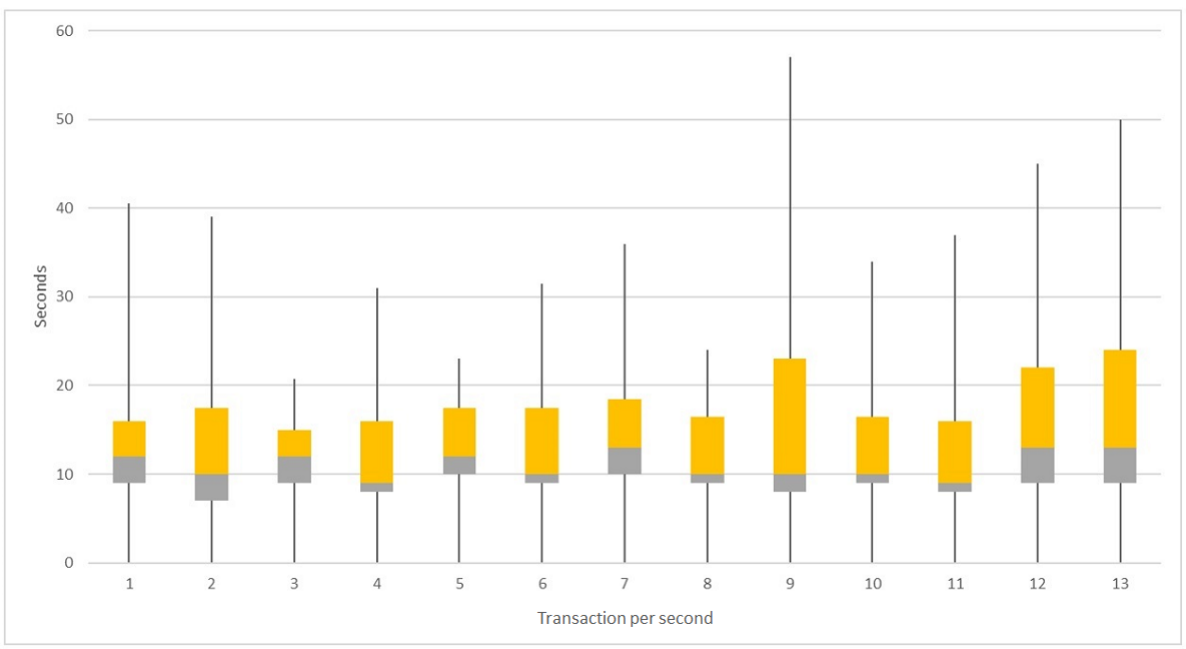
Box plot of simulation results for 1 to 13 transactions per second groups. The boxes show the different quantiles of time cost related to different scales of transactions per second.

In this study, the scalability of the blockchain using various transaction frequencies from 1 to 14 TPS through blockchain adapters was tested. Figure 5 shows the time spent granting permission from different scales (the 14 TPS group was excluded because of incomplete results). Once the permission is granted by writing the transactions into blocks, the receiver can retrieve the metadata from the smart contract through the blockchain adapter without making another transaction for users to validate the legitimacy. This means that the average time of receiving metadata is much shorter than the grant permission. The script of the 9 TPS group runs slowly compared with the former groups. All the blockchain nodes were restarted separately, and the script was restarted with the 10 TPS group. The speed is affected by the processing speed of the blockchain nodes and Ethereum performance. The starting node’s blockchain adapter was used to control the overall frequency. All transactions from the blockchain adapters of health care facility nodes will queue in the starting node’s adapter until the earlier batches of transactions have been executed by each blockchain adapter. We controlled the overall frequency as 13 TPS, which avoids the Ethereum’s scalability constraints by spacing the transactions.

### Subject Recruitment for Clinical Trials

To prove the compatibility of our layered blockchain system, another application was implemented as subject recruitment for clinical trials. This application involves posting criteria, granting permission, and data exchange. To use this application, each clinical trial sponsor needs to provide at least one blockchain node with a blockchain adapter installed. All patients who want to participate in clinical research are required to opt into the application so that they can receive the recruitment criteria. This application involves the following procedures:

- Clinical research recruiters will send recruitment criteria to the opted-in patients in the *interfacing layer*. The criteria also contain the recruiters’ blockchain address used for patient authentication.
- Patients who received the criteria can check whether they are matched. If they are interested in clinical research, they can authenticate the clinical research recruiter to access their EHR.
- Recruiters will be notified by the blockchain of whether any patients have granted their permissions to access their data. Then, they will retrieve patient data for precise matching.
- The recruiters will notify the patients if they are matched and send further instructions.

**Figure 6:**
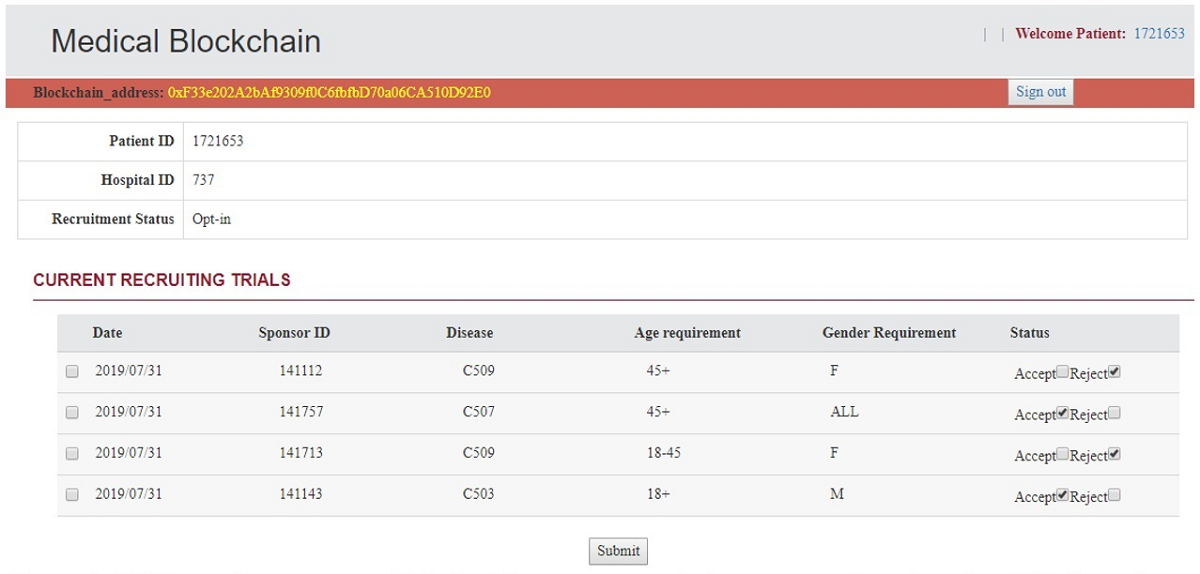
Graphical user interface for patients to permit clinical trial sponsors to access their data. The graphical user interface shows the patient’s blockchain address, opt-in status, current recruiting trials’ basic recruitment criteria, and permission options. When the patients select the recruiting trial and click submit, the sponsor will be added to their access list in the USER manager smart contract through the blockchain adapter.

We have implemented a GUI to demonstrate the usage of this application. Patients need to opt for this application to receive the current clinical trial recruitment information. We randomly selected 4 sponsors to post their recruitment criteria using their blockchain adapters through the blockchain *interfacing layer*. Only patients who opted will receive recruiting clinical trial information. Figure 6 shows the GUI for a simulated patient with ID 1721653. It will provide basic information for the patient, such as blockchain address and registered health care facility ID. The recruiting criteria include the sponsor ID, basic inclusion criteria such as age and gender, and study disease (in this case, we used the primary site for the study disease in our simulation). Patients can use the GUI to grant the sponsors access to receive their data to have a precise match with their clinical trial criteria. This action will automatically send a transaction to the *user manager* smart contract to add the sponsor’s blockchain address to their access list. Through our simulation, 2 sponsors successfully received permission in an average of 3.07 seconds.

## Discussion

### Conclusions and Future Work

This paper described an augmented layered blockchain system in development for most health care applications involving data coordination across multiple health care facilities. The design of this layered architecture provides generic functions and methods for application developers to securely collect data from different sources without requiring extensive experience of the blockchain technology. The layered architecture allows users the ability to audit the legitimacy of previously occurring transactions but prevents users from modifying any components in the blockchain. The features of blockchain provide a solution to current data coordination challenges. The blockchain-based approach extends the ownership of the EHR data set to each patient. On the basis of decentralized features of blockchain technology for peer-to-peer transactions, this approach can greatly reduce the health care data set sign-off and release. Data security and authenticity are also guaranteed by the immutability of the blockchain and smart contract–regulated data exchange.

Through our simulation process, our system empirically proved the feasibility of the architecture for health care applications. We also tested the scalability of our blockchain system and provided an optimal solution to avoid blockchain scalability constraints. Our future work will continue to evaluate the validation mechanism to improve the blockchain performance and add an AI component on the *application layer* for data analytics to maximize the use of EHRs based on the layered blockchain system.

### Limitation

The main limitation of our approach is the setup requirement from each participating site. Each health care facility is required to provide at least one blockchain node to the system and keep an encrypted EHR outside of the operation EHR for registered patients into the secured data stores that can communicate with blockchain adapters. Patients will potentially also need to provide blockchain nodes such as mobile devices to exchange and store their personal health records generated by their personal medical devices. The performance of the model can be affected by the properties of the blockchain node. If a single node creates a mass of transactions at the same time through the blockchain adapter, this action will use up all the memory and break the node before it is sent to the blockchain.

## Abbreviations

AI: artificial intelligence
API: application program interface
EHR: electronic health record
GUI: graphical user interface
HIE: health information exchange
MOST: Ministry of Science and Technology in Taiwan
PoW: Proof of Work
TPS: transactions per second
